# Characteristics of and reasons for patients with chronic obstructive pulmonary disease to continue smoking, quit smoking, and switch to heated tobacco products

**DOI:** 10.18332/tid/142848

**Published:** 2021-11-01

**Authors:** Kuniaki Hirai, Akihiko Tanaka, Tetsuya Homma, Tomoko Kawahara, Naruhito Oda, Hatsuko Mikuni, Yoshitaka Uchida, Haruhisa Saito, Yosuke Fukuda, Akiko Fujiwara, Yoko Sato, Tomoki Uno, Hideki Inoue, Shin Ohta, Fumihiro Yamaguchi, Shintaro Suzuki, Tsukasa Ohnishi, Hironori Sagara

**Affiliations:** 1 Division of Respiratory Medicine and Allergology, Department of Medicine, Showa University School of Medicine, Tokyo, Japan; 2 Division of Respiratory Medicine, Department of Medicine, Yamanashi Red Cross Hospital, Yamanashi, Japan; 3 Division of Respiratory Medicine, Department of Medicine, Kokuho Asahi Chuo Hospital, Chiba, Japan; 4 Division of Respiratory Medicine, Department of Medicine, Ebara Hospital, Tokyo, Japan; 5 Division of Respiratory Medicine, Department of Medicine, Odawara Municipal Hospital, Odawara, Japan; 6 Division of Respiratory Medicine, Department of Medicine, Showa University Fujigaoka Hospital, Fujigaoka, Japan

**Keywords:** chronic obstructive pulmonary disease, cigarettes, smoking, heated tobacco products

## Abstract

**INTRODUCTION:**

Smoking is the leading cause of chronic obstructive pulmonary disease (COPD), and smoking cessation is the most effective treatment for patients with COPD. However, few studies have investigated the continuation/cessation of smoking and heated tobacco products (HTP) in patients with COPD. The objective of this study was to examine the characteristics of patients with COPD, those who are current smokers and those who switched from cigarettes to HTP, and to examine the reason for the continuation or cessation of smoking.

**METHODS:**

This multicenter, cross-sectional study included 411 outpatients with COPD. Data for this study were part of a study conducted for a comprehensive evaluation of the smoking status and clinical factors in patients with COPD and their families.

**RESULTS:**

Logistic regression analysis revealed that a younger age, longer duration of smoking, fewer daily cigarettes, and lower modified Medical Research Council (mMRC) dyspnea score, and a lower Simplified Nutritional Appetite Questionnaire (SNAQ) score for appetite, were characteristics of current smokers (age OR=0.94; duration of smoking OR=1.07; number of cigarettes per day OR=0.94; mMRC OR=0.68; SNAQ OR=0.83; p<0.05). The logistic regression analysis model showed that a younger age and higher education level were associated with the use of HTP (age OR=0.83; higher education level OR=4.63; p<0.05). Many of the current smokers displayed smoking behaviors that are not guaranteed to be safe, such as reducing smoking or switching to lighter cigarettes or HTP.

**CONCLUSIONS:**

Patients with COPD who continue smoking tended to have low appetite as well as smoking behaviors that are not guaranteed to be safe. Physicians should provide appropriate guidance to these patients on smoking cessation.

## INTRODUCTION

Chronic obstructive pulmonary disease (COPD) causes many deaths worldwide, with studies reporting smoking to be the leading cause of COPD^1-3^. Smoking cessation is the most effective treatment for patients with COPD, since it can reduce the rate of decline in lung function, reduce hospitalization rates, and improve the quality of life^4-7^. Although several studies have investigated the clinical characteristics of patients with COPD who continue to smoke and who quit smoking^8-10^, few studies have investigated the reasons why these patients quit or not quit smoking.

Recently, the availability of novel types of nicotine/ tobacco products, such as electronic cigarettes (e-cigarettes) and heated tobacco products (HTP), has changed Japanese smoking practices^11,12^. The perception of a reduced health risk compared to smoking cigarettes has been reported among the reasons for selecting a specific HTP brand in Japan^13^. Although there are several reports stating that HTP and e-cigarettes may reduce the exposure to harmful substances, compared to cigarettes^14^, quitting smoking altogether is preferred due to reported adverse health effects resulting from the use of HTP and e-cigarettes^15-17^. One report advocated not recommending these products as a replacement for nicotine to patients who were smokers with underlying chronic respiratory diseases such as COPD, since the mechanisms of damage could facilitate the underlying immune impairment and exacerbate complications or disease progression^15^. However, few reports have examined the characteristics of patients with COPD who substituted conventional cigarettes with HTP. Therefore, we conducted a multicenter, cross-sectional study to clarify the following: 1) the characteristics of patients with COPD who are current smokers and past smokers, 2) the smoking status and reasons for continued smoking in patients with COPD who are current smokers, 3) the characteristics of patients with COPD who substituted conventional cigarettes with HTP, and 4) the reasons for quitting smoking in past smokers who have COPD.

## METHODS

### Study design and patients

Data for this study were obtained as part of a multicenter, cross-sectional study conducted at six facilities in four prefectures in Japan between November 2017 and May 2020 that comprehensively evaluated patients with COPD and their families’ smoking status, satisfaction, and understanding of the disease. The participants were outpatients, and questionnaires were randomly distributed at the discretion of the outpatient doctor. Among the patients who had previously smoked, those who answered that they had quit smoking and had not smoked at all for longer than one month were defined as past smokers, while those who answered that they were currently smoking were defined as current smokers^18^. We also confirmed on the questionnaire whether the patient was using HTP. The diagnosis of COPD was made based on the Global Initiative for Chronic Obstructive Lung Disease criteria: a post-bronchodilator ratio^4^ of forced expiratory volume in one second to forced vital capacity <0.7 and a clinical diagnosis of COPD made by a respiratory physician. The inclusion criteria were: age ≥40 years and a smoking history. The exclusion criteria were: acute exacerbation of COPD within 4 weeks, concomitant respiratory diseases other than COPD, concurrent active malignancy, and an inability to read or understand questionnaires. A total of 108 patients were excluded due to the following reasons: the questionnaire could not be collected for various reasons (n=59), there was a discrepancy in the answers to the questions regarding tobacco (n=43), and there was a large discrepancy in the answers regarding clinical items other than tobacco (n=6). Although we have data on the number of questionnaires distributed and collected by physicians, the exact number of patients with COPD who were treated by physicians at outpatient clinics was unclear; therefore, the actual total number of patients with COPD visiting our study facilities was unknown. Finally, this study included 411 outpatients with COPD. All participants provided written informed consent. This study was conducted in accordance with the Declaration of Helsinki guidelines and was approved by the ethics committee of the relevant institution as well as an independent ethics committee.

### Questionnaire contents and investigated clinical factors

The participants in this study received a questionnaire, which was returned to the outpatient physician upon completion. The duration of smoking and the number of cigarettes smoked per day were confirmed. For current smokers including HTP users, a survey was conducted on smoking behavior, reasons for not quitting smoking, and the content of guidance on smoking cessation that was provided by an outpatient doctor. For past smokers, the reason for quitting smoking and whether they regretted smoking were confirmed using the questionnaire. One family member, identified by the patient as their closest, was evaluated and classified as either a current smoker, past smoker, or a never smoker.

Data on the education level of patients were collected and the level classified as lower than high school, high school, vocational school, or university. The patients’ most recent respiratory function test results and body mass index (BMI: kg/m^2^) measurements were extracted from the clinical records. Patient health status was assessed using the COPD Assessment Test (CAT)^19^, dyspnea was evaluated using the modified Medical Research Council (mMRC) dyspnea scale^20^, appetite was measured using the Simplified Nutritional Appetite Questionnaire (SNAQ)^21^, and mood disorders using the Hospital Anxiety and Depression Scale -Anxiety (HADS-A) and HADS-Depression (HADS-D)^22^. A higher score for CAT, mMRC, HADS-A, and HADS-D indicated that the patient had more severe symptoms. A high SNAQ score was indicative of an increased appetite.

### Statistical analysis

Continuous variables that were normally distributed in the histogram were expressed as mean ± standard deviation, while the non-normally distributed variables were expressed as median and interquartile range. Student’s t-test or the Mann-Whitney U test was used for continuous variables. The χ^2^ and Fisher’s exact test were used for categorical variables.

Logistic regression analysis was performed to identify the clinical factors associated with current smokers and patients who switched to HTP. Age, sex, and clinical variables were selected as covariates for multivariable analysis. In performing multivariable analysis, education level was divided into two groups: lower than school and high school, and vocational school and university. Multivariable analysis was conducted for factors with a p≤0.2 in the univariate analysis. In the multivariable analysis including more factors whose p-value in the univariate analysis was >0.2, the duration of smoking and number of cigarettes smoked per day were analyzed separately from the smoking pack-years. All statistical tests were two-tailed, and p<0.05 was considered to indicate statistical significance. All statistical analyses were performed using JMP Pro for Macintosh version 14 (SAS Institute, Cary, NC).

## RESULTS

### Patient background and clinical features of current smokers

Table 1 shows the patient background data. A total of 411 patients with COPD and a mean age of 73.8±8.4 years were enrolled in this study. The number of current smokers was 82 (20.0%), the average duration of smoking was 39.5±12.2 years, and the average number of cigarettes smoked per day was 25.9±13.0. The median CAT score, and mean mMRC score and predicted %FEV_1_ were 14 (8–20), 1.4±1.1, and 64.5±23.4%, respectively. The proportion of current smokers in the families of participating patients with COPD was 11%.

**Table 1:** Characteristics of patients included in the study (N=411)

*Patients*	*Mean±SD, n (%) or Median (25th–75th percentile)*
Age (years)	73.8±8.4
Sex (male)	362 (88.1)
Current smoker	82 (20.0)
Duration of smoking (years)	39.5±12.2
Cigarettes smoked per day	25.9±13.0
Smoking pack-years	50.4±28.9
CAT score	14 (8–20)
mMRC score	1.4±1.1
SNAQ score	15 (14–16)
HADS-A score	4 (2–7)
HADS-D score	6 (4–9)
BMI (kg/m^2^)	22.7±3.9
FEV_1_ (L)	1.6 ± 0.7
%FEV_1_ (%pred)	64.5±23.4
Education level: Lower than high school/high school/vocational school/university (%)	23.5/42.2/6.1/28.2
Living alone	95 (23.1)
Smoking rate among family members (%)	11.0

CAT: chronic obstructive pulmonary disease assessment test. mMRC: modified medical research council. SNAQ: Simplified Nutritional Appetite Questionnaire. HADS-A: Hospital Anxiety and Depression Scale - Anxiety. HADS-D: Hospital Anxiety and Depression Scale - Depression. BMI: body mass index. FEV_1_: forced expiratory volume in 1 second. %FEV_1_: percent predicted forced expiratory volume in the first second.

Table 2 shows the clinical characteristics of the patients with COPD who were current and past smokers. Current smokers have been smoking for longer (45.5±11.2 vs 38.0±12.0 years, p<0.01) and smoked fewer cigarettes per day (20.3±11.4 vs 27.3±13.0, p<0.01) than past smokers. Current smokers had milder symptoms of shortness of breath (mMRC: 1.2±0.9 vs 1.5±1.1, p=0.04), had less of an appetite (SNAQ: 14^13-15^ vs 15^14-16^, p<0.01), were more usually living alone (34.2% vs 20.4%, p<0.01), and had more current smokers in their family (26.7% vs 7.9%, p<0.01) than past smokers. There was no difference between the two groups regarding age, sex, CAT score, HADS-A score, HADS-D score, respiratory function, or education level.

**Table 2:** Characteristics of patients with COPD who are current and past smokers (N=411)

*Patients*	*Current smokers (n=82) Mean±SD, n (%) or Median (25th–75th percentile)*	*Past smokers (n=329) Mean±SD, n (%) or Median (25th–75th percentile)*	*p*
Age (years)	72.4±8.4	74.1±8.4	0.11
Sex (male)	71 (86.6)	291 (88.5)	0.70
Duration of smoking (years)	45.5±11.2	38.0±12.0	<0.01
Number of cigarettes smoked per day	20.3±11.4	27.3±13.0	<0.01
Smoking pack-years	45.9±27.7	51.5±29.1	0.11
CAT score	12.5 (8.75–18.25)	14 (8–20)	0.64
mMRC score	1.2±0.9	1.5±1.1	0.04
SNAQ score	14 (13–15)	15 (14–16)	<0.01
HADS-A score	5 (2–7)	4 (2–7)	0.72
HADS-D score	6 (3–9.5)	6 (4–8)	0.40
BMI (kg/m^2^)	22.4±4.0	22.8±3.8	0.42
FEV_1_ (L)	1.7±0.6	1.6±0.7	0.30
%FEV_1_ (%pred)	66.6±22.5	64.0±23.6	0.20
Education level: Lower than high school/high school/vocational school/university (%)	25.4/40.8/4.2/29.6	23.0/42.6/6.5/27.9	0.87
Living alone	28 (34.2)	67 (20.4)	<0.01
Smoking rate among family members (%)	26.7	7.9	<0.01

CAT: chronic obstructive pulmonary disease assessment test. mMRC: modified medical research council. SNAQ: Simplified Nutritional Appetite Questionnaire. HADS-A: Hospital Anxiety and Depression Scale - Anxiety. HADS-D: Hospital Anxiety and Depression Scale - Depression. BMI: body mass index. FEV_1_: forced expiratory volume in 1 second. %FEV_1_: percent predicted forced expiratory volume in the first second.

Logistic regression analysis was performed to evaluate the clinical factors associated with current smoking (Supplementary file Table S1). A younger age, longer duration of smoking, fewer number of cigarettes smoked per day, lower mMRC score, and lower SNAQ score were characteristics of current smokers after multivariable analysis: age (OR=0.94; 95% CI: 0.91–0.98, p<0.01); duration of smoking (OR=1.07; 95% CI: 1.04–1.11, p<0.01); number of cigarettes per day (OR=0.94; 95% CI: 0.91–0.97, p<0.01); mMRC (OR=0.68; 95% CI: 0.50–0.92, p=0.01); and SNAQ (OR=0.83; 95% CI: 0.71–0.97, p=0.02; Supplementary file Table S1). Living alone was not found to be significant after multivariable analysis but tended to be associated with current smoking (OR=1.71; 95% CI: 0.94– 3.14, p=0.08).

### Smoking behavior, reasons for continuing smoking, guidance on smoking cessation, and characteristics of HTP users in current smokers

Current smokers were surveyed on smoking behaviors that applied to them: 61 (74.4%) answered ‘I reduced smoking’, 40 (48.8%) answered ‘I switched to a light cigarette’, and 16 (19.5%) answered ‘I switched to HTP’ (Supplementary file Table S2). The most common reason for not quitting smoking was ‘I want to quit smoking but I have a desire to smoke’ (n=55; 67.1%), the second most common reason was ‘I reduced smoking or switched to HTP’ (n=23; 28.0%), and the third most common reason was ‘I don't think smoking cessation will be effective anymore’ (n=15; 18.3%) (Supplementary file Table S2). Table 3 shows the characteristics of the patients whose reason for continuing smoking was to reduce their amount of smoking or having switched to HTP. These patients were young (p<0.01) and had a poor HADS-A score (p=0.01). The guidance on smoking cessation that current smokers received from outpatient doctors was: 25 (30.5%) answered ‘instructed to quit smoking every time’, 32 (39.0%) answered ‘instructed to quit smoking occasionally’, and 18 (22.0%) answered ‘almost no smoking cessation guidance given or smoking status not confirmed’ (Supplementary file Table S2).

**Table 3:** Characteristics of the patients based on whether their reason for continuing to smoke was due to smoking reduction or switching to heated tobacco products, or another reason (N=82)

*Characteristics*	*Due to smoke reduction or switching to HTP (n=23) Mean±SD, n (%) or Median (25th–75th percentile)*	*Not due to smoke reduction or switching to HTP (n=59) Mean±SD, n (%) or Median (25th–75th percentile)*	*p*
Age (years)	68.0±8.0	74.0±8.0	<0.01
Sex (male)	19 (82.6)	52 (88.1)	0.51
Duration of smoking (years)	44.1±10.9	46.1±11.3	0.46
Number of cigarettes smoked per day	23.3±14.4	19.2±10.0	0.15
Smoking pack-years	53.0±38.6	43.1± 21.8	0.15
CAT score	11 (7.5–17.5)	14 (9–18.5)	0.83
mMRC score	1.0±0.6	1.3±1.0	0.34
SNAQ score	14 (12–15)	14 (13–15)	0.70
HADS-A score	6 (4–8)	4 (2–6.3)	0.01
HADS-D score	5 (3–9)	7 (3.8–11)	0.12
BMI (kg/m^2^)	20.9±4.4	22.5±4.0	0.09
FEV_1_ (L)	1.8±0.7	1.6±0.6	0.35
%FEV_1_ (%pred)	67.0±20.4	66.4±23.4	0.92
Living alone	7 (30.4)	21 (35.6)	0.66
Smoking prevalence among family members (%)	25.0	27.3	0.88
**Education level** (%)			0.22
Lower than high school	18.2	28.6	
High school	31.8	44.9	
Vocational school	9.1	2.0	
University	40.9	24.5	

CAT: chronic obstructive pulmonary disease assessment test. mMRC: modified medical research council. SNAQ: Simplified Nutritional Appetite Questionnaire. HADS-A: Hospital Anxiety and Depression Scale - Anxiety. HADS-D: Hospital Anxiety and Depression Scale - Depression. BMI: body mass index. FEV_1_: forced expiratory volume in 1 second. %FEV_1_: percent predicted forced expiratory volume in the first second.

Table 4 shows the clinical characteristics of the 16 patients who were using HTP and the 66 patients who were not. HTP users were younger (p<0.01) and more highly educated (p=0.01), although no significant differences were found for other clinical factors. Three logistic regression analysis models were constructed to evaluate the clinical factors associated with patients using HTP (Table 5). Even after performing multivariable analysis, a younger age was associated with HTP users, while a higher education level tended to be associated with HTP users (Table 5).

**Table 4:** Characteristics of patients with COPD who switched from smoking cigarettes to using heated tobacco products (HTP), (N=82)

*Characteristics*	*HTP users (n=16) Mean±SD, n (%) or Median (25th–75th percentile)*	*Non-HTP users (n=66) Mean±SD, n (%) or Median (25th–75th percentile)*	*p*
Age (years)	64.9±8.6	74.2±7.3	<0.01
Sex (male)	15 (93.8)	56 (84.9)	0.35
Duration of smoking (years)	42.3±9.2	46.3±11.5	0.20
Number of cigarettes smoked per day	21.9±10.6	20.0±11.7	0.55
Smoking pack-years	48.0±32.6	45.3±26.6	0.73
CAT score	13 (7–19)	12 (9–18)	0.92
mMRC score	1.1±0.8	1.2±1.0	0.51
SNAQ score	14 (13–15)	14 (13–15)	0.86
HADS-A score	6 (4–7.8)	4 (2–7)	0.13
HADS-D score	4.5 (3–7)	6 (4–11)	0.12
BMI (kg/m^2^)	22.0±4.4	22.5±4.0	0.66
FEV_1_ (L)	1.9±0.6	1.6±0.6	0.16
%FEV_1_ (%pred)	65.2±18.5	66.9±23.5	0.80
Education level: Lower than high school/high school/vocational school/university (%)	0/33.3/6.7/60.0	32.1/42.9/3.6/21.4	0.01
Living alone	5 (31.2)	23 (34.9)	0.79
Smoking rate among family members (%)	22.2	27.8	0.74

CAT: chronic obstructive pulmonary disease assessment test. mMRC: modified medical research council. SNAQ: Simplified Nutritional Appetite Questionnaire. HADS-A: Hospital Anxiety and Depression Scale - Anxiety. HADS-D: Hospital Anxiety and Depression Scale - Depression. BMI: body mass index. FEV_1_: forced expiratory volume in 1 second. %FEV_1_: percent predicted forced expiratory volume in the first second.

**Table 5:** Unavailable and multivariable logistic regression analysis for factors of patients who switched to using heated tobacco products

*Variable*	*Univariate analysis*		*Multivariate analysis 1*		*Multivariate analysis 2*		*Multivariate analysis 3*	
	*OR (95% CI)*	*p*	*OR (95% CI)*	*p*	*OR (95% CI)*	*p*	*OR (95% CI)*	*p*
Age (years)	0.86 (0.79–0.94)	0.01	0.83 (0.73–0.94)	<0.01	0.83 (0.73–0.95)	<0.01	0.86 (0.77–0.96)	<0.01
Sex (male)	2.68 (0.32–22.6)	0.37			2.38 (0.15–36.6)	0.52	2.15 (0.15–31.5)	0.56
Duration of smoking	1.03 (0.98–1.09)	0.20	1.03 (0.94–1.15)	0.38	1.03 (0.94–1.13)	0.38		
Number of cigarettes	0.99 (0.94–1.03)	0.55			1.03 (0.97–1.11)	0.33		
smoked per day								
Smoking pack-years	1.00 (0.98–1.02)	0.73					1.00 (0.98–1.03)	0.71
Highly educated (vocational school/university)	6.00 (1.75–20.6)	<0.01	4.58 (1.13–21.4)	0.03	5.08 (0.96–26.8)	0.06	4.88 (0.94–25.3)	0.05
CAT score	1.00 (0.93–1.09)	0.92			1.00 (0.84–1.20)	0.96	1.01 (0.85–1.20)	0.89
mMRC score	1.24 (0.66–2.32)	0.51			0.99 (0.38–2.90)	0.98	1.04 (0.36–2.95)	0.95
HADS-A score	1.21 (0.91–1.63)	0.13	1.19 (0.95–1.54)	0.17	0.82 (0.61–1.10)	0.17	1.19 (0.63–1.12)	0.22
%FEV_1_	1.00 (0.98–1.03)	0.80			1.00 (0.96–1.03)	0.86	0.99 (0.95–1.03)	0.58

Three multivariate analyses were performed by information on smoking: Multivariate analysis 1 (duration of smoking); Multivariate analysis 2 (duration of smoking and number of cigarettes smoked per day); and Multivariate analysis 3 (smoking pack-years). CAT: chronic obstructive pulmonary disease assessment test. mMRC: modified medical research council. HADS-A: Hospital Anxiety and Depression Scale - Anxiety. %FEV_1_: percent predicted forced expiratory volume in the first second.

### Reasons for quitting smoking and views on past smoking by past smokers

The reasons for quitting smoking by past smokers were: 122 (37.1%) answered ‘because I was diagnosed with a respiratory disease’, 79 (24.0%) answered ‘because I had difficulty breathing’, 72 (21.9%) answered ‘because I was instructed by a doctor’, 50 (15.2%) answered ‘because my family told me to quit’, and 6 (1.8%) answered ‘because the price of cigarettes went up’ (Supplementary file Table S3).

In response to the question ‘what would you like to do if you knew you had a lung disease and could go back to the past before you started smoking?’, the participants provided the following answers: 117 (35.5%) answered ‘if I could go back in time, I wouldn't have smoked a single cigarette’, and 145 (44.1%) answered ‘if I could go back in time, I would have stopped when I was young’ (Supplementary file Table S3).

## DISCUSSION

Our study demonstrates the following clinically important points. First, the characteristics of current smokers among patients with COPD tended to be a younger age, longer smoking duration, smoking fewer cigarettes per day, experiencing less shortness of breath (dyspnea), and a lower appetite. Second, many of the current smokers tended to make changes to their smoking behaviors, such as reducing smoking or switching to lighter cigarettes or HTP that have not yet been proven scientifically safe. Third, the characteristics of patients with COPD who switched to HTP tended to be a younger age and higher education level.

Our study showed that 20% of patients with COPD continued to smoke and demonstrated the clinical characteristics of current smokers. Compared with previous studies, the rate of current smoking in patients with COPD in this study was low^12,23-25^. The reason for this low rate may be that patients who visited the department of respiratory medicine were more likely to be health-conscious and that the physician had already provided smoking cessation guidance to them. In multivariable analysis, current smokers were younger, had smoked for a longer time, smoked fewer cigarettes, experienced lesser shortness of breath, and had a lower appetite when compared to past smokers. This was a cross-sectional study; therefore, we cannot draw conclusions about causal relationships. However, in this study, many current smokers had reduced smoking, which may be the reason for the low number of cigarettes smoked per day and the long smoking duration in current smokers. Previous studies have shown that a reduction in smoking does not provide a sufficient reduction in health risks^26,27^; therefore, appropriate guidance on smoking cessation is needed for patients who continue to smoke due to the misconception that reduction or switching to HTP is sufficient. The poorer mMRC score in past smokers compared to current smokers was consistent with previous studies in which patients quit smoking when their respiratory symptoms worsened^28,29^ . In fact, the second most common reason for past smokers quitting smoking in this study was ‘because I had difficulty breathing’. We have shown that among patients with COPD, current smokers tend to have a poorer appetite than past smokers. The results of this study are considered reasonable, as nicotine affects the central nervous system mechanism of the hypothalamus that is related to satiety and smoking is thought to reduce the patient’s appetite^30^. Previous studies targeting the general population have shown that smoking cessation causes weight gain^31,32^, and this study showed that patients with COPD were expected to have an improved appetite as a result of smoking cessation. A low BMI is indicative of a poor prognosis in patients with COPD^33^, and Asian patients with COPD have a lower BMI than Western patients^34,35^. Therefore, smoking cessation, which can be expected to improve appetite and lead to weight gain, is especially important in Asian patients with COPD. Univariable analysis showed a significant difference between current and past smokers who are living alone, but multivariable analysis showed no significant difference. Among the past smokers, some patients answered that they quit smoking because they were instructed to quit by their families, and patients living alone or smoking with their families tended to be at risk of continuing smoking. This suggests that families influence smoking behavior among patients with COPD.

Many current smokers tended to make changes to their smoking behaviors, such as reducing smoking or switching to lighter cigarettes or HTP that have not yet been proven to be scientifically safe^17,26,27,36^. In recent years, several studies have suggested that HTP and e-cigarettes have adverse health effects^17,37-41^, while in this study, approximately 20% of smokers used HTP. The characteristics of patients with COPD who switched to HTP tended to be of younger age and higher education level. The reason for HTP use being common in younger patients is that it is predominantly spread among young patients, as shown in a previous study in Japan^42^. The patients whose reason for continuing smoking was to reduce their amount of smoking or having switched to HTP, tended to have high level of anxiety. Therefore, we speculate that their behavior of reducing smoking or switching to HTP could be related to their anxiety. We need more information on the risks of smoking reduction, light tobacco use, and HTP use in patients who smoke.

The diagnosis of respiratory diseases, awareness of symptoms of respiratory problems, and guidance from doctors accounted for 83% of the reasons for quitting smoking, indicating that medical factors play a major role in quitting smoking. Previous studies have shown that the diagnosis of COPD is associated with smoking cessation behavior^43,44^, and the results of this study are consistent with those findings. Previous studies have shown that guidance by doctors is useful even if the guidance is simple^45^, and this study also showed that guidance on smoking cessation is important for patients with COPD. This study did not include data on whether the change in smoking behavior occurred before or after the COPD diagnosis. Therefore, if the smoking behavior was exclusively investigated after the COPD diagnosis, the proportion of COPD diagnoses contributing to the smoking behavior could have been even greater. The fact that approximately 70% of the patients in this study did not receive guidance on smoking cessation at every consultation indicates that inadequate guidance on smoking cessation is provided to patients with COPD. This may be due to pulmonologists feeling powerless with regard to convincing current smokers to stop smoking^46^.

### Limitations

This study has several limitations. First, carbon monoxide levels in exhaled breath were not measured and the smoking status was based on self-report. Second, the primary medical institution was not included and the total number of patients with COPD who were treated at the institutions participating in our study was unclear; therefore, there is a possibility that selection bias may have occurred. Third, the section of the questionnaire on smoking that was used in this study has not been previously validated. Based on the above limitations, a larger longitudinal study among patients with COPD is required in the future.

## CONCLUSIONS

The characteristics of patients with COPD who were current smokers tended to be a lower average number of daily cigarettes smoked, longer smoking duration, younger age, less shortness of breath, and lower appetite than patients who were past smokers. In this study, HTP were used by approximately 20% of patients with COPD who were current smokers, and its use was especially common in younger and highly educated patients. Many patients displayed smoking behaviors that are not guaranteed to be safe, such as smoking reduction and using HTP; therefore, medical professionals should provide appropriate education on smoking cessation.

## Data Availability

The data supporting this research are available from the authors on reasonable request.
