# Supplementary material for: Characteristics of and reasons for patients with chronic obstructive pulmonary disease to continue smoking, quit smoking, and switch to heated tobacco products
Source: Tob Induc Dis. 2021 Nov 1;19:85. doi: 10.18332/tid/142848 (PMC8562318; doi:10.18332/tid/142848)
Supplement: Supplementary file 1 [file TID-19-85-s1.pdf]

Supplementary Table S1. Univariable and multivariable logistic regression analysis for factors of patients with COPD who are current smokers

	**Univariate analysis**			**Multivariate analysis**		
	Odds ratio	95% CI	*p*-value	Odds ratio	95% CI	*p*-value
Age	0.98	0.95–1.01	0.11	0.94	0.91–0.98	<0.01
Sex (male)	1.19	0.58–2.44	0.64	1.19	0.53–2.66	0.68
Duration of smoking	1.06	1.04–1.09	<0.01	1.07	1.04–1.11	<0.01
Number of cigarettes smoked per day	0.94	0.92–0.97	<0.01	0.94	0.91–0.97	<0.01
mMRC score	0.77	0.60–0.98	0.03	0.68	0.50–0.92	0.01
SNAQ score	0.82	0.71–0.94	<0.01	0.83	0.70–0.97	0.02
Living alone	2.03	1.20–3.44	<0.01	1.71	0.94–3.14	0.08

CI, confidence interval; mMRC, Modified Medical Research Council; SNAQ, Simplified Nutritional Appetite Questionnaire

Supplementary Table S2. Survey for current smokers

Did you change your smoking content?	
I reduced smoking	61 (74.4%)
I switched to a light cigarette	40 (48.8%)
I switched to heated tobacco products	16 (19.5%)

Reasons for not quitting smoking	
I want to quit smoking but I have a desire to smoke	55 (67.1%)
I reduced smoking or switched to heated tobacco products	23 (28.0%)
I don't think smoking cessation will be effective anymore	15 (18.3%)
I have family who smoke	7 (8.5%)
I have a colleague who smokes	5 (6.1%)

Guidance on smoking cessation	
Instructed to quit smoking every time	25 (30.5%)
Instructed to quit smoking occasionally	32 (39.0%)
Almost no smoking cessation guidance is given	9 (11.0%)
Smoking status is not confirmed	9 (11.0%)
I tell my doctor that I am quitting smoking	7 (8.5%)

Supplementary Table S3. Survey for past smokers

**The reasons for quitting smoking**	
Because I was diagnosed with a respiratory disease	122 (37.1%)
Because I had difficulty breathing	79 (24.0%)
Because I was instructed by a doctor	72 (21.9%)
Because my family told me to quit	50 (15.2%)
Because the price of cigarettes went up	6 (1.8%)

**What would you like to do if you knew you had a lung disease and could go back to the past before you started smoking?**	
If I could go back in time, I wouldn't have smoked a single cigarette	117 (35.5%)
If I could go back in time, I would have stopped when I was young	145 (44.1%)
If I could go back in time, I think I would have continued to smoke	67 (20.4%)

© 2021 Hirai K. et al.
